# Advanced Multi-Objective Optimization for Laser Cladding of H13 Die Steel with CFOA

**DOI:** 10.3390/ma18071617

**Published:** 2025-04-02

**Authors:** Tianlu Liu, Ruichen Wang, Bin Han, Rui Wang

**Affiliations:** School of Mechanical Engineering, Shijiazhuang Tiedao University, Shijiazhuang 050043, China; ltl17633330094@163.com (T.L.); ruichen.wang@stdu.edu.cn (R.W.); h03200418@163.com (B.H.)

**Keywords:** H13 die steel, multi-objective optimization in laser cladding, CFOA algorithm, analytic hierarchy process (AHP)

## Abstract

The quality of laser cladding is strongly influenced by process parameters, which interact in complex and often nonlinear ways. The existing literature primarily focuses on the influence of process parameters on surface properties. However, limited research has explored the relationship between process parameters, surface properties, and their optimization. To bridge this gap, this study introduces a novel parameter modeling and optimization approach using the Catch Fish Optimization Algorithm (CFOA). Key process parameters, including laser power, scanning speed, and powder feeding rate, were systematically analyzed for their effects on the surface quality of H13 die steel. An orthogonal experimental design was employed to develop a regression model capable of accurately predicting cladding quality metrics, such as dilution rate, microhardness, and aspect ratio. To address the multi-objective nature of the optimization problem, the analytic hierarchy process (AHP) was used to transform it into a single-objective framework. The proposed approach identified an optimal parameter combination: laser power of 1628.19 W, scanning speed of 9.9 mm/s, and powder feeding rate of 14.73 g/min. Experimental validation demonstrated significant improvements in cladding performance, with enhancements of 19.71% in dilution rate, 3.37% in microhardness, and 28.66% in aspect ratio. The CFOA also shows global search capabilities and precision compared to conventional methods, making it a robust tool for complex optimization tasks. This study presents an innovative methodology for optimizing laser cladding processes, providing effective H13 die steel repair solutions. It also emphasizes the versatility of metaheuristic algorithms for advancing manufacturing process optimization.

## 1. Introduction

Laser cladding represents a cutting-edge surface treatment technology, where a high-energy laser beam is utilized to melt both the cladding material and the substrate. The process results in rapid solidification, forming a coating that exhibits exceptional bonding strength with the substrate [1,2,3]. This method can fabricate high-performance coatings with hardness, wear, and corrosion resistance [4,5,6]. Due to these advantages, this technology has been widely utilized in material surface modification treatments [7,8], biomedical fields [9,10,11], aerospace applications (such as turbine blade repair [12] and compressor reinforcement [13]), and transportation equipment (including automotive [14] and shipbuilding [15]). It is also employed in parts repair (gears [16], rails [17], and turbine blades [18]) for enhancing surface properties, extending components’ service life, and reducing production costs.

Heavy-duty components are often required to function under extreme loads and harsh environmental conditions in industries like large-scale construction machinery and aerospace. Many critical components sustain damage due to surface scratches, spalling, cracking, pitting, localized fractures, and corrosion, leading to material degradation over time [19,20,21]. Custom-designed and non-standardized components pose considerable difficulties in repair, manufacturing, or replacement, often leading to equipment downtime and significant losses in operational efficiency [22]. Direct disposal or recycling of such critical parts results in extensive resource wastage, conflicting with the principles of sustainable and green manufacturing [23,24]. Laser cladding provides a viable solution to repair damaged components while aligning with sustainable manufacturing principles. Its ability to restore functionality and extend service life reduces material waste and operational downtime.

Repairing and reinforcing failed key components has become an essential strategy for advancing the circular economy and fostering the sustainable development of the equipment manufacturing sector. Laser cladding has become a leading technology for surface modification, component repair, and remanufacturing owing to its precise process control, minimal thermal influence on materials, and compatibility with a wide range of materials [25,26,27]. H13 steel is extensively employed in key components due to its mechanical properties and thermal resistance [28]. However, its high alloy content, sensitivity to thermal cracking, and reliance on precise process parameter control present significant challenges when using laser cladding technology [29,30]. In this paper, H13 was utilized to investigate the optimization of process parameters for laser cladding of H13 steel to improve manufacturing efficiency and product quality. Laser cladding enables precise repair and surface enhancement of H13 steel components, which are critical for high-stress applications. This process enhances hardness and wear resistance, ensuring prolonged service life.

The quality, microstructure, mechanical properties, and geometry of the cladding layer are profoundly influenced by key process parameters, including laser power, scanning speed, powder feed rate, and laser beam diameter [31,32,33,34,35,36]. Inappropriate parameter settings often lead to severe defects, such as porosity and cracks, which compromise the overall quality and functionality of the cladding [37,38,39]. The single-pass, single-layer cladding technique is the foundation for multi-pass multi-layer cladding and directly determines the overall quality of the cladding area [40]. As a result, comprehensive analysis and optimization of single-pass single-layer cladding are essential to achieving process outcomes and ensuring high-quality repairs and modifications [41,42].

Conventional methodologies, including trial-and-error approaches, Taguchi methods, and response surface methods (RSMs), heavily depend on experimental design and the expertise of practitioners to adjust process parameters. For example, Dong and Guo et al. [43,44] employed RSM to optimize the geometric characteristics and performance of laser cladding coatings, achieving substantial improvements in hardness, flatness, and surface quality. Similarly, Yu and Lian et al. [45,46] integrated Taguchi methods with grey relational analysis (GRA) to refine both multi-track and single-track laser cladding processes, resulting in improved coating width, height, and dilution rate, along with improved efficiency and morphological consistency. Yue et al. [47] combined RSM with GRA to optimize a multi-objective cobalt-based alloy cladding process, significantly improving cross-sectional morphology and microstructural properties. Although notable successes have been achieved, conventional approaches are fundamentally constrained by the boundaries of experimental design and the limited number of trials available. Such constraints often lead to parameter selections that deviate from optimal solutions. The challenges mentioned above are particularly evident in complex, multi-objective scenarios, where intricate interactions among parameters require more comprehensive and robust optimization strategies to unlock the full potential of laser cladding processes.

The advancement of artificial intelligence (AI) technology has facilitated the creation of sophisticated algorithms that highlight efficiency and accuracy in laser cladding research. For instance, Li and Zhang et al. [48,49] combined support vector regression (SVR) with genetic algorithms, including A-NSGA-III and NSGA-II, to optimize laser cladding processes, significantly improving cladding layer properties. Shu et al. [50] applied a multi-objective grey wolf optimization algorithm (MOGWO) with the response surface methodology (RSM) to refine powder ratios and geometric morphology, improving mechanical properties and microstructural integrity. Additionally, Du et al. [51] used the hybrid whale optimization algorithm (CWOA) and the AHP-CV weighting method to transform multi-objective problems into single-objective optimization frameworks, improving coating properties.

Advancements in process parameter optimization for laser cladding have addressed several challenges, yet significant issues remain unresolved. Conventional methods, such as the Taguchi and response surface methodology (RSM), rely heavily on experimental design, confining optimization results to predefined experimental conditions. Such approaches also face difficulties effectively handling complex multivariate nonlinear problems, often producing solutions that deviate from the optimum. Modern intelligent algorithms have enhanced optimization efficiency; however, practical limitations persist, including inadequate model generalization and high sensitivity to initial conditions.

This study introduces an optimization method for laser cladding processes to overcome existing challenges based on the Catch Fish Optimization Algorithm (CFOA). CFOA represents a novel metaheuristic approach that integrates the advantages of global exploration and local refinement, offering a robust solution to intricate optimization problems. Its capability for comprehensive search minimizes the likelihood of becoming trapped in local optima, ensuring more reliable results. The quality of a single-layer cladding is strongly influenced by key process parameters, including laser power (*P*), scanning speed (*V*), and powder feeding speed (*F*) [52,53,54]. Laser power governs the extent of the powder and substrate melting, scanning speed determines the interaction duration between the laser and material, and powder feeding speed impacts efficiency and cost-effectiveness [55]. The assessment of cladding layer quality typically involves three primary indicators: microhardness (*HV*), dilution rate (*η*), and aspect ratio (*W/H*). Microhardness reflects the material’s hardness characteristics, dilution rate measures the strength of the metallurgical bond between the cladding layer and substrate, and aspect ratio affects the geometric structure and overall stability of the cladding layer.

This paper is structured in four main sections. Section 1 explores the laser cladding experiments, examining the effects of process parameters on quality indicators such as microhardness, dilution rate, and aspect ratio. Section 2 focuses on developing a regression model combined with the analytic hierarchy process (AHP) to quantify relationships between parameters and performance metrics. Section 3 introduces the Catch Fish Optimization Algorithm (CFOA) for process parameter optimization, incorporating adjustments to meet practical engineering requirements. Section 4 validates the normalization as a fundamental step in preprocessing experimental data. It standardizes data values to the optimization framework through experimental trials, demonstrating significant improvements in cladding performance. The conclusion summarizes the findings and highlights the study’s contributions to laser cladding optimization.

## 2. Experimental Setup

### 2.1. Equipment and Configuration

**Figure 1:**
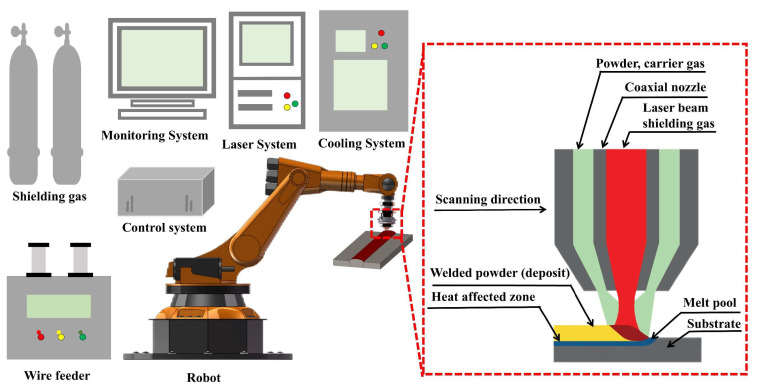
Schematic diagram of laser cladding equipment and coaxial powder-feeding system.

The laser cladding system used in this study consists of a coaxial powder-feeding laser head, an RC-PGF-D powder feeder, an IPG-3000W carbon dioxide laser, and a precision worktable. A protective environment was maintained using 99% pure argon gas to minimize oxidation during cladding. The laser spot diameter was set at 3 mm, and the shielding gas flow rate was adjusted to 10 L/min to maintain consistent process stability and quality. This configuration enables precise control over the cladding parameters, facilitating the production of high-quality coatings. A schematic representation of the laser cladding system is provided in Figure 1, highlighting its key components and operational framework.

The cladding substrate was a rectangular specimen of H13 Mold steel, measuring 120 mm × 100 mm × 8 mm in thickness. Before the experiment, the substrate underwent mechanical polishing with a 120-mesh alumina grinding wheel to remove the oxide layer and expose the metallic luster. The surface was then ultrasonically cleaned with acetone (40 kHz, 15 min), acid-washed with 5% hydrofluoric acid (120 s, room temperature), and rinsed with deionized water to remove residual oxides and contaminants. Finally, ultrasonic testing verified the substrate’s integrity, ensuring no internal defects such as cracks, inclusions, voids, delamination, or debonding. The temperature of the substrate was controlled at 25 ± 2 °C before the experiment to avoid the disturbance caused by the ambient temperature.

H13 metal powder was selected as the cladding material due to its compatibility with the substrate and excellent metallurgical properties. To improve fluidity and minimize the risk of macroscopic defects such as pores or cracks in the cladding layer, the powder was dried at 130 °C for 2 h before use. Drying was crucial in preventing moisture-related inconsistencies and ensuring uniform deposition during cladding. The chemical compositions of the H13 substrate and metal powder are provided in Table 1, highlighting suitability for high-performance laser cladding applications.

### 2.2. Experimental Design and Methodology

A three-factor four-level orthogonal design (Table 2) was adopted to evaluate parameter effects on dilution rate, microhardness, and aspect ratio. In the multi-parameter coupling process of laser cladding, the interactions between process parameters (particularly the synergistic thermal effects between laser power and scanning speed, and the dynamic equilibrium between powder feed rate and energy density) exhibit significant nonlinear characteristics that critically influence clad quality. This study successfully identifies threshold behaviors of these interactions through orthogonal experimental design, which effectively captures equilibrium distribution patterns using limited experimental trials.

**Figure 2:**
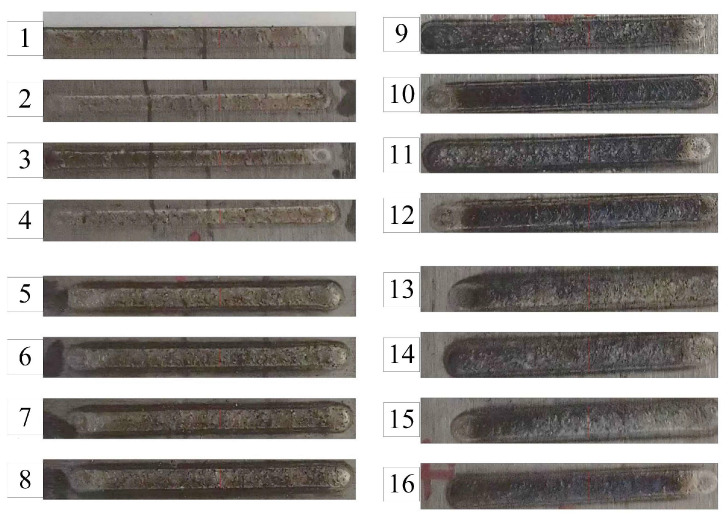
Surface morphology of single-pass cladding layer obtained by laser cladding orthogonal test.

**Figure 3:**
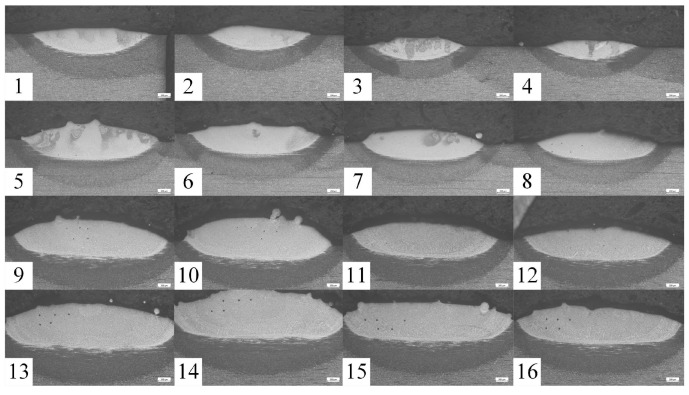
Macromorphology of single-pass cladding cross-section obtained by laser cladding orthogonal test.

A three-factor, four-level orthogonal design assessed parameter effects and variable interactions on critical quality indicators: microhardness, dilution rate, and aspect ratio. Parameter ranges were established through dual validation integrating literature analysis and experimental calibration. Initial windows were defined based on critical evaluation of H13 steel laser cladding studies, with feasibility confirmed through macroscopic morphology and microhardness screening, ultimately yielding a stable parameter set for orthogonal experiments. The specific parameters and levels used in the orthogonal experiment are detailed in Table 2, offering a structured representation of the experimental design. Visual representations of the cladding layer’s surface and macrostructure are shown in Figure 2 and Figure 3, respectively. The framework allows for a balanced evaluation of each factor, supporting robust statistical analysis and optimization of process parameters to enhance cladding performance. 

To achieve a high-quality cladding layer, dilution rate, microhardness, and aspect ratio are selected as critical evaluation indicators due to their essential role in determining overall cladding performance. These metrics provide a comprehensive assessment of the cladding’s mechanical, metallurgical, and geometrical characteristics, facilitating the optimization of process parameters. The dilution rate reflects the degree to which the base material melts and mixes with the molten coating. A lower dilution rate minimizes mixing the base material with the molten cladding material, ensuring that the cladding layer retains properties closely aligned with the added material [56]. However, low values can lead to insufficient bond strength between the cladding layer and the substrate, increasing the risk of spalling or delamination under mechanical stress. Preliminary experiments suggest that a dilution rate of approximately 30% achieves an optimal balance between metallurgical bonding strength and the preservation of the cladding material’s inherent properties.

**Figure 4:**
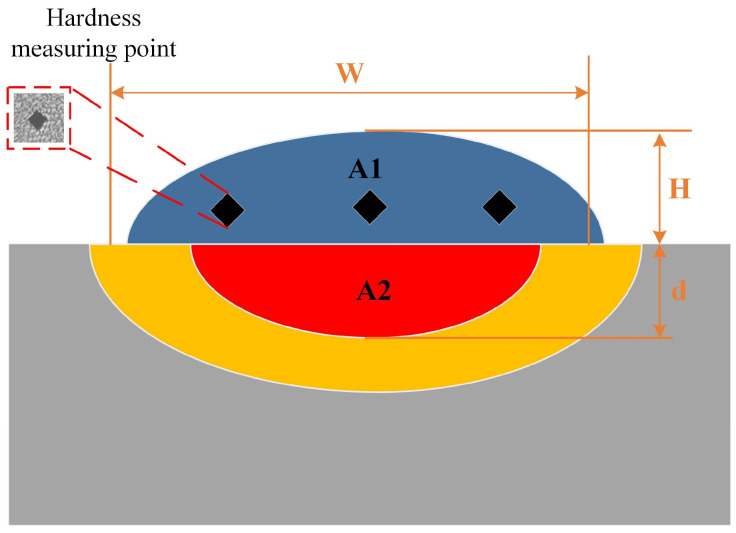
Schematic diagram of the cross-section of the cladding layer.

Figure 4 shows the cross-sectional structure of a single-layer laser cladding sample, which comprises four distinct zones: the cladding layer, the molten pool, the heat-affected zone, and the substrate. The geometric dimensions of the molten pool are denoted as width (*W*), height (*H*), and depth (*d*). The cladding layer and molten pool areas are *A* and *B*, respectively. The dilution rate is quantitatively determined using Equation (1), measuring the degree of mixing between the substrate and the cladding material.

(1) $$\eta (\%)=\frac{A_{2}}{A_{1}+A_{2}}=\frac{d}{H+d}\times 100\%$$

The *HV* microhardness is a critical parameter that reflects the coating’s mechanical properties. Higher microhardness values correlate with increased coating hardness, significantly improving wear resistance and durability. These properties are essential for ensuring the performance and longevity of the cladding layer in demanding applications.

The aspect ratio (W/H) provides valuable information about the molten pool’s shape and the coating deposition’s efficiency. An increased aspect ratio results in a wider molten pool, improving the cladding layer’s coverage and deposition efficiency [57]. However, a high aspect ratio can compromise surface quality and weaken the metallurgical bond between the cladding layer and the substrate. Preliminary experiments identified an optimal aspect ratio of approximately 3, balancing efficient deposition with structural integrity. Optimizing the dilution rate, microhardness, and aspect ratio effectively improves the quality of the cladding layer. Such optimization enhances stability and reliability, ensuring suitability for practical engineering applications [58].

A single cladding layer was produced using the laser cladding process, and a region with visual characteristics was selected for analysis. The assigned area was cut into a specimen measuring 10 mm × 10 mm × 10 mm using a metallographic cutting machine. The specimen was polished sequentially with sandpaper ranging from 200# to 1500# and refined with abrasive polishing paste using an automatic abrasive polishing machine to achieve a smooth and reflective surface. The polished sample was then rinsed with anhydrous ethanol and dried thoroughly. Corrosion treatment was performed using a 4 vol% nitric alcohol solution until the cladding layer’s boundary became distinctly visible. The microstructure of the cladding layer was then examined using an Axio Vert. A1 metallurgical microscope, revealing its detailed morphology. The Vickers hardness of the cladding layer was measured using an HVS-1000A digital micro-Vickers hardness tester (HSS GROUP, Shenzhen, China), applying a 500 g load for 15 s. Five consecutive measurements were taken at 1.5 mm intervals across each sample to ensure reliability, and the average value was calculated. The hardness data are summarized in Table 3. Visual representations of the cladding layer’s surface and macrostructure are shown in Figure 2 and Figure 3, respectively.

### 2.3. Influence of Process Parameters on Cladding Properties

**Figure 5:**
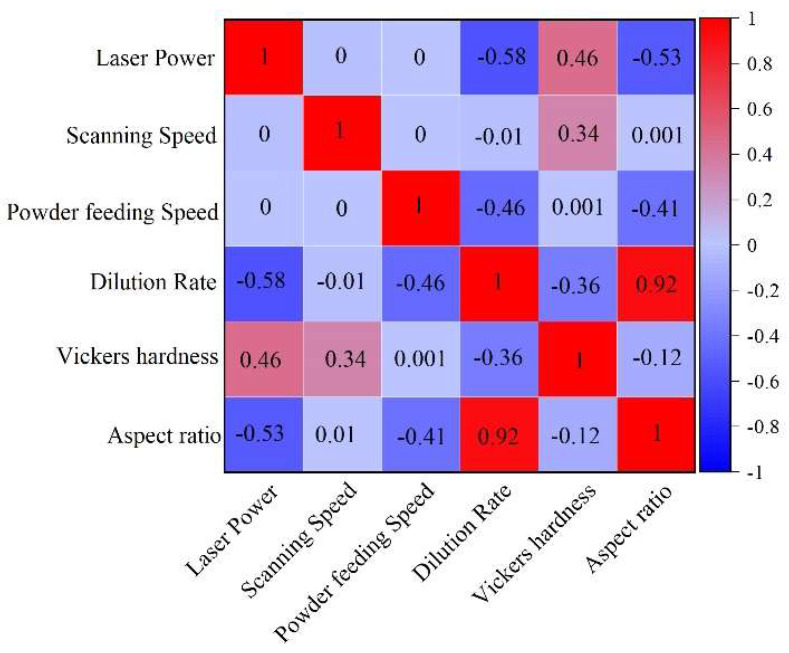
Correlation matrix of process parameters and target response values.

Figure 5 shows the effects of laser power, scanning speed, and powder-feeding speed on the cladding layers’ dilution rate, Vickers hardness, and aspect ratio. Laser power demonstrates a significant influence on cladding properties, showing a strong positive correlation with Vickers hardness (+0.46), a pronounced negative correlation with the dilution rate (−0.58), and a moderate negative correlation with the aspect ratio (−0.53). The findings highlight laser power as a crucial parameter for regulating the cladding layer’s material properties and geometric characteristics, making it a key variable to monitor and refine for process optimization.

Powder-feeding speed substantially affects the geometry and dilution rate of the cladding layer. A moderate negative correlation with the dilution rate (−0.46) and a positive correlation with the aspect ratio (+0.41) suggest that increasing the powder-feeding speed improves the geometric structure of the cladding. However, its negligible impact on hardness (correlation of 0.0) indicates that powder-feeding speed is secondary in enhancing material strength.

Scanning speed exerts minimal influence on the evaluated cladding properties. The weak correlation with Vickers hardness (+0.34) and negligible effects on dilution rate (−0.01) and aspect ratio (0.0) suggest that scanning speed is not a dominant factor in optimizing cladding performance. The results indicate the importance of carefully regulating laser power and powder-feeding speed to balance material properties and geometric precision in cladding.

**Figure 6:**
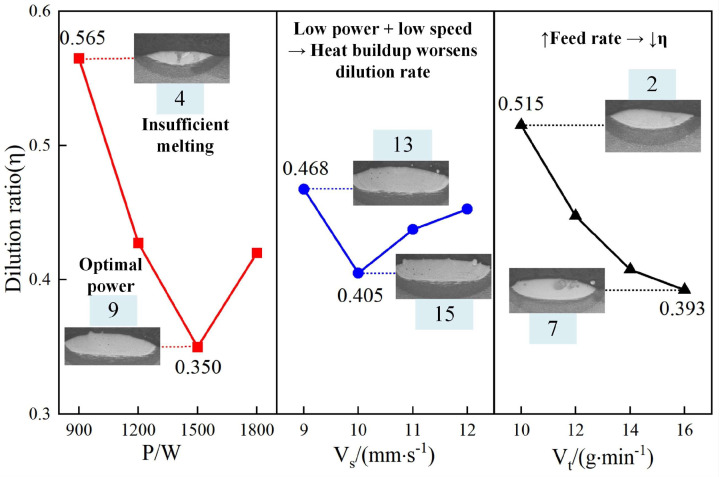
Effect of process parameters on dilution rate.

**Figure 7:**
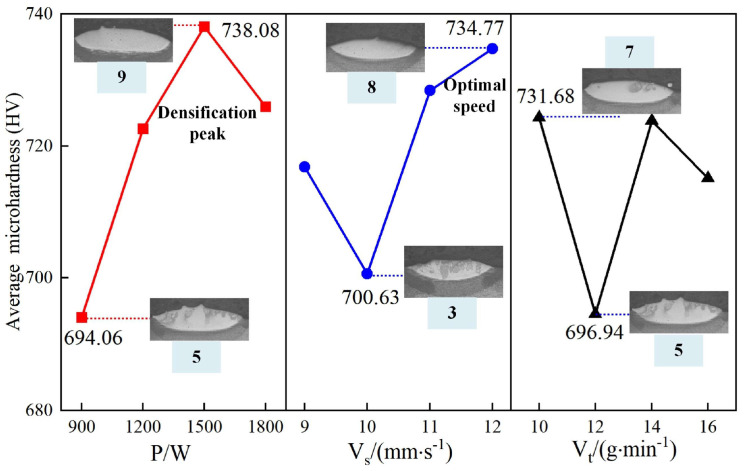
Effect of process parameters on aspect ratio.

**Figure 8:**
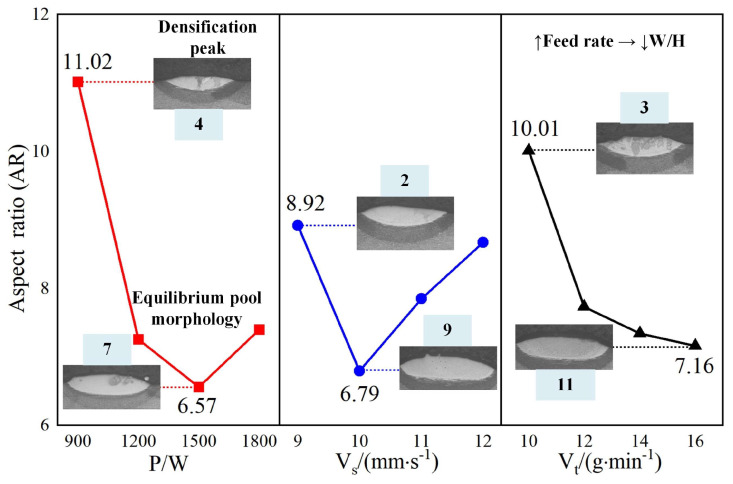
Aspect ratio main effect plot.

The findings from Figure 5 provide a foundational understanding of how key process parameters influence cladding properties, which are further explored in Figure 6, Figure 7 and Figure 8 to examine the effects on the dilution rate, highlighting critical thresholds for optimal performance. Figure 6 presents the primary effect plot for the dilution rate of the cladding layer, illustrating the impact of laser power, powder feed rate, and scanning speed. Among the evaluated factors, laser power exerts the most significant influence on the dilution rate, followed by powder feed rate, with scanning speed having the least impact.

At 900 W laser power, the dilution rate reaches its maximum of 0.565 (No. 4). As laser power increases to 1500 W, the dilution rate decreases to a minimum of 0.35 (No. 9). Further increasing the power to 1800 W causes the dilution rate to rise again to 0.42. The results suggest that insufficient laser power fails to adequately melt both the substrate and the powder, leading to a higher dilution rate. In contrast, excessive laser power can over-melt the substrate, resulting in an elevated dilution rate. Optimizing laser power is essential to balance effective melting and minimizing unwanted substrate incorporation into the cladding layer.

An increase in powder feed rate leads to a continuous reduction in dilution rate, reaching a minimum of 0.3925 (No. 7) at a feed rate of 16. The increased amount of powder entering the melt pool raises the powder-to-substrate ratio and reduces substrate dilution. A sufficient powder feed rate fills the melt pool more effectively, lowering the dilution rate. However, excessive powder feed rates can result in incomplete melting of the powder within the melt pool, negatively affecting the cladding layer’s quality and performance.

The impact of scanning speed on dilution rate is relatively minor but follows a distinct pattern. The dilution rate initially decreases and then increases, reaching a minimum of 0.405 (No. 15) at a scanning speed of 10. At lower scanning speeds, the laser remains in contact with the substrate for longer, melting a more significant portion of the substrate and increasing the dilution rate. As scanning speed increases, the interaction time shortens, reducing the dilution rate. However, if the scanning speed becomes too high, the melt pool may not fully form, causing the dilution rate to rise again. This indicates the need for precise control over scanning speed to maintain cladding quality.

Figure 7 presents the principal effect plot for the microhardness of the cladding layer, highlighting the influence of laser power, scanning speed, and powder-feeding speed. Laser power has the most pronounced impact on microhardness, followed by scanning speed, with powder-feeding speed exerting the least effect.

An increase in laser power initially raises microhardness, which declines beyond a certain threshold. The highest microhardness of 738.08 HV (No. 9) is observed at 1200 W, while the lowest value of 694.06 HV (No. 5) occurs at 900 W. Moderate laser power promotes densification of the cladding layer, enhancing hardness. Insufficient laser power results in incomplete melting of the powder and substrate, reducing microhardness. In contrast, excessive laser power can cause overheating, leading to grain growth in the cladding layer and negatively affecting hardness.

Scanning speed exhibits a distinct V-shaped relationship with microhardness. The peak value of 734.77 HV (No. 8) is achieved at a scanning speed of 12, with the lowest value of 700.63 HV (No. 3) occurring at 10. Lower scanning speeds increase the interaction time between the laser and substrate, promoting densification of the cladding layer despite higher heat accumulation. Higher scanning speeds reduce heat buildup by limiting the laser’s interaction time, thereby improving hardness through better structural preservation.

Powder-feeding speed most effectively impacts microhardness at a moderate rate. A low feeding rate leads to an insufficient powder-to-substrate ratio within the molten pool, limiting densification and reducing hardness. Extremely high feeding rates can result in incomplete powder melting, creating an uneven microstructure and adversely affecting hardness.

Figure 8 presents the primary effect plot for the aspect ratio of the cladding layer, highlighting the influence of laser power, powder feed rate, and scanning speed. Laser power indicates the most significant impact, followed by the powder feed rate, while scanning speed has the least influence. The aspect ratio reaches a maximum value of 11.02 (No. 4) at 900 W laser power, decreasing to a minimum of 6.56 (No. 7) at 1500 W before rising again. This suggests that lower laser power generates a wider cladding layer due to insufficient energy to create a deep molten pool. In contrast, higher laser power reduces the cladding width while increasing the molten pool depth, resulting in a higher aspect ratio. Optimizing laser power is essential for achieving a balanced cladding geometry.

For the powder feed rate, the aspect ratio consistently declines, with a maximum of 10.01 (No. 3) and a minimum of 7.16 (No. 11). Higher powder feed rates introduce more significant quantities of powder into the molten pool, causing the cladding layer’s height to increase while its width remains relatively constant. This imbalance reduces the aspect ratio, highlighting the need for precise control of the powder feed rate to maintain the desired cladding geometry.

Scanning speed has a less pronounced effect on the aspect ratio but follows a predictable pattern. Lower scanning speeds result in extended laser interaction with the material, producing a wider cladding layer due to the complete melting of the substrate and powder. At higher scanning speeds, the reduced interaction time limits molten pool formation, leading to a narrower cladding layer and a corresponding increase in the aspect ratio.

The analysis of Figure 5, Figure 6, Figure 7 and Figure 8 demonstrates the varying effects of laser power, powder feed rate, and scanning speed on the critical properties of the cladding layer. Laser power is the most influential parameter, significantly affecting dilution rate, microhardness, and aspect ratio, while powder feed rate and scanning speed play secondary but complementary roles. Proper adjustment of laser power is essential to balance melting efficiency, densification, and cladding geometry. Powder feed rate primarily affects layer uniformity and geometry, with excessive rates potentially causing incomplete melting. While less influential, scanning speed offers a means for fine-tuning by adjusting interaction time and heat input. The results indicate the necessity of balancing parameters to ensure consistent and high-quality cladding layers suitable for engineering applications.

## 3. Data Processing

### 3.1. Data Normalisation

Normalization is a fundamental step in preprocessing experimental data. It standardizes data values to a consistent scale, removing dimensional inconsistencies and enabling effective feature comparison. The process improves the performance of machine learning algorithms, ensures numerical stability, and simplifies model interpretation and data visualization [49]. Due to the distinct range of experimental values and the need to maintain relationships between variables, Min-Max normalization is applied. The Min-Max normalization technique scales data to a specified range while preserving proportional relationships among variables. To align with the optimization goal of deviation minimization, the dilution rate is normalized to a target value of 0.3, and the aspect ratio is adjusted to 3. Vickers hardness is scaled to ensure all values remain positive, with higher values reflecting better outcomes. The normalized dataset provides a robust foundation for subsequent analysis and is summarized in Table 4.

### 3.2. Mathematical Modeling for Performance Prediction

The performance indicators of the cladding layer, namely *f*_1_ (dilution rate), *f*_2_ (Vickers hardness), and *f*_3_ (aspect ratio), are treated as response variables for predictive modeling. Regression analysis is used to develop mathematical models for each response variable, expressed as functions of the input parameters: *A* (laser power), *B* (powder feed rate), and *C* (scanning speed).

The regression model for predicting the dilution rate (*f*_1_) is derived as follows:

$$\begin{array}{ll} f_{1} & =2.617-0.223A-1.191B-0.592C-0.1047A^{2}+0.627B^{2}+0.04844C^{2}\;-0.2219AB\\ & +0.0344AC-0.0792B^{3}+0.0563A^{2}B+0.0312A^{2}C \end{array}$$

The analysis of variance (ANOVA) results for the dilution rate fitting model are summarized in Table 5. The coefficient of determination (*R*^2^) is 99.66%, indicating that the model explains nearly all the observed dilution rate variations, highlighting its exceptional predictive capability. The F-value for the regression model is 105.46, with a corresponding *p*-value of 0.000, well below the standard significance threshold of 0.05. It confirms the statistical significance of the model and its ability to effectively describe the observed variation in dilution rate. The sum of squares of the error term (Seq SS) is 0.002773, accounting for only 0.34% of the total variation. The minimal value indicates that the model captures nearly all the variation, leaving an insignificant portion attributed to error. In addition, the mean square of the error term (Adj MS) is 0.000693, further validating the model’s precision and reliability.

The fitting result for predicting microhardness (*f*_2_) is expressed in the following regression model:

$$\begin{array}{c} f_{2}=-3.33-0.471A+2.409B+3.822C-0.066A^{2}-1.214B^{2}-1.345C^{2}+0.482AB\\ -0.1816AC-0.0997BC+0.0544A^{3}+0.1553B^{3}+0.1722C^{3}-0.1015A^{2}B \end{array}$$

Table 6 presents the analysis of variance results for the microhardness model. The model’s significance level is 0.091, slightly above the standard threshold of 0.05, indicating moderate explanatory power. Among the variables, *B* (powder feed rate) and *C* (scanning speed) show significance levels below 0.05, confirming their strong influence on microhardness. The F-value analysis shows that *C* has the most substantial impact, followed by *B*, while *A* (laser power) has a relatively minor effect, with a *p*-value of 0.585.

The lack of fit is not statistically significant, as indicated by a *p*-value greater than 0.05, suggesting that the model adequately represents the experimental data. The coefficient of determination (*R*^2^) is 0.9854, showing that the model accounts for 98.54% of the variation in the experimental results. The minimal difference between the adjusted *R*^2^ and predicted *R*^2^ further supports the model’s predictive accuracy and reliability, demonstrating its suitability for describing the relationship between process parameters and microhardness.

The fitting result for the width-to-height ratio (*f*_3_) is expressed in the following regression model:

$$\begin{array}{c} f_{3}=4.132-1.702A-1.224B-1.514C+0.665A^{2}+0.493B^{2}+0.332C^{2}-0.296AB\\ +0.0837AC+0.1556BC-0.0749A^{3}-0.0791B^{3}-0.0379C^{3}+0.0666AB^{2} \end{array}$$

Table 7 summarizes the analysis of variance results for the width-to-height ratio model, showing that the model is statistically significant with a *p*-value of 0.046. Among the variables, *A* (laser power) has the most significant impact on the aspect ratio, with a *p*-value of 0.041. The *p*-value for C (scanning speed) is 0.057, close to the significance threshold, indicating a moderate contribution. The *p*-value for *B* (powder feed rate) is 0.124, suggesting a weaker influence.

For the quadratic term *A*^2^, the *p*-value of 0.085 suggests a marginal effect, approaching the threshold for statistical significance. The interaction term *A* × *C* shows a notable impact, with a *p*-value of 0.046, revealing the combined influence of laser power and scanning speed on the aspect ratio.

The *R*^2^ value of 0.9356 indicates that the model accounts for 93.56% of the variation in the experimental data. The difference between the adjusted *R*^2^ and predicted *R^2^* is 0.1321, confirming the model’s ability to fit the data well. The findings suggest that the model effectively captures the relationship between process parameters and aspect ratio, providing a reliable predictive tool.

**Figure 9:**
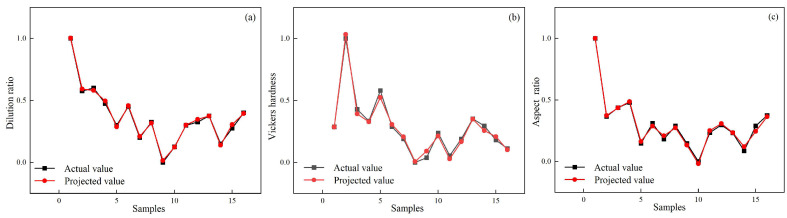
Comparison of predicted and experimental (**a**) dilution rate, (**b**) Vickers hardness, and (**c**) aspect ratio.

**Figure 10:**
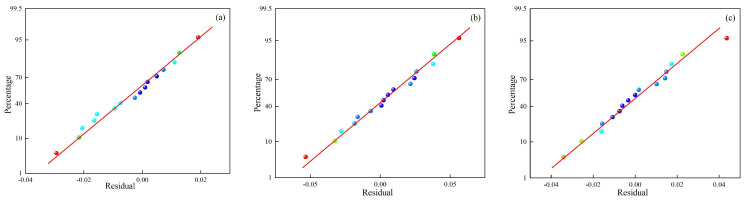
Normal distribution of residuals’ (**a**) dilution rate, (**b**) Vickers hardness, and (**c**) aspect ratio.

**Figure 11:**
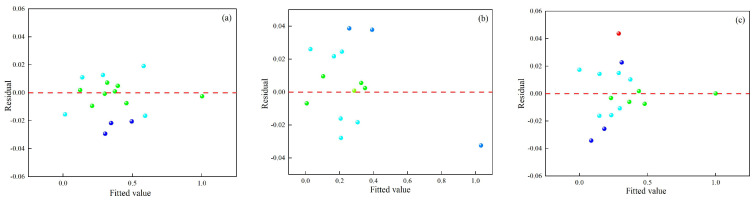
Residual plots of (**a**) dilution rate, (**b**) Vickers hardness, and (**c**) aspect ratio.

Figure 9a, Figure 10a and Figure 11a compare predicted and experimental values, revealing a high degree of alignment and a consistent trend. The close agreement demonstrates the accuracy of the prediction model in representing the experimental data and capturing variations in the results. Minor deviations observed between predicted and experimental values highlight the robustness of the model’s predictive ability while confirming its effectiveness in handling complex data relationships. Figure 9b, Figure 10b and Figure 11b present the normal distribution of residuals from the model. Data points align closely along a straight line, indicating that the residuals show a normal distribution and verify the absence of systematic prediction errors, providing strong evidence of the reliability and stability of the model. The adherence to normality confirms the validity of statistical assumptions underlying the regression analysis. Figure 9c, Figure 10c and Figure 11c show the residual plots, showing data points cantered around zero without any identifiable pattern. The random distribution of residuals confirms that errors are independent of the predicted values, indicating that the model does not systematically overestimate or underestimate outcomes. The randomness supports the model’s calibration and ability to provide unbiased predictions across experimental conditions.

The comprehensive analysis of predicted values, residual distributions, and error randomness validates the model’s strong predictive performance. The ability to align closely with experimental data and the absence of systematic biases confirms the robustness and reliability of the developed prediction model for practical applications.

### 3.3. Weight Determination Using the Analytic Hierarchy Process (AHP)

**Figure 12:**
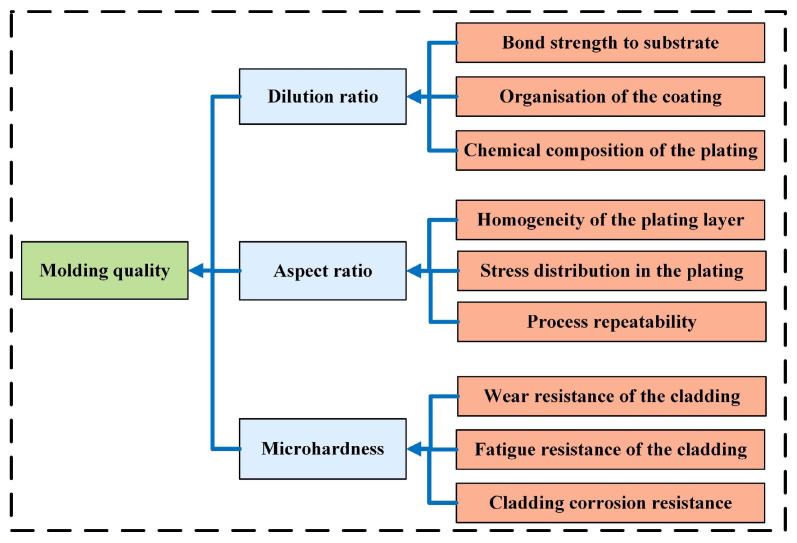
AHP structure diagram.

In Figure 12, the analytic hierarchy process (AHP) was applied to systematically evaluate the influence of dilution rate, microhardness, and aspect ratio on molding quality. It allows for an objective assessment of the relative importance of these factors, providing a quantitative basis for optimizing process parameters in production settings. The derived weights assigned to each factor serve as theoretical guidance for achieving high-quality molding results.

Incorporating expert knowledge and the team’s extensive experience in laser cladding ensured the accuracy and reliability of the AHP analysis. Questionnaires were distributed to gather input on scale values, which were assigned using a combination of expert opinions and empirical methods. The mean values derived from this process formed the basis of the judgment matrix.

Table 8 presents the judgment matrix used for the analysis. It offers a structured representation of the pairwise comparisons among the three factors. It facilitates the calculation of relative weights, offering a robust and consistent evaluation of the factors influencing molding quality.

The equation for calculating weights is as follows:

(2) $$W=\left(\begin{array}{c} \frac{W_{1}}{\sum_{i=1}^{3}W_{i}}\\ \frac{W_{2}}{\sum_{i=1}^{3}W_{i}}\\ \frac{W_{3}}{\sum_{i=1}^{3}W_{i}} \end{array}\right)$$

where *W* represents the normalized weights for each factor; W_1_, W_2_, and W_3_ denote the individual weights of the factors before normalization.

The individual weight for each factor is calculated as:

(3) $$W_{i}=\frac{1}{3}\sum_{j=1}^{3}A_{ij}^{\prime }$$

where *W_i_* indicates the weight of the *i*th factor, and $A_{ij}^{\prime }$ represents the normalized value of the *i*th factor relative to the *j*th factor.

The normalized value is determined using:

(4) $$\;A_{ij}^{\prime }=\frac{a_{ij}}{\sum_{k=1}^{3}a_{ij}}$$

where *a_ij_* stands for the pairwise comparison value of the *i*th factor relative to the *j*th factor, and $\sum_{k=1}^{3}a_{ij}$ denotes the sum of all pairwise comparison values in the *j*th column.

Finally, the calculated weights are:

(5) $$W=\left(\begin{array}{c} 0.3184\\ 0.3996\\ 0.2820 \end{array}\right)$$

The model for evaluating the quality of molding is expressed as:

(6) $$R=\left(\begin{array}{c} 0.3184\\ 0.3996\\ 0.2820 \end{array}\right)\left(\begin{array}{ccc} f_{1} & f_{2} & f_{3} \end{array}\right)$$

To summarize the findings of Section 3, the analysis focuses on the impact of process parameters on cladding quality through data normalization, predictive modeling, and weight determination. Min-Max normalization standardizes data to ensure consistent scaling, facilitating accurate comparisons and reliable predictions. Regression models for dilution rate, microhardness, and aspect ratio exhibit substantial accuracy and statistical significance, validated through residual and variance analyses. The analytic hierarchy process effectively assesses the relative importance of performance indicators by integrating expert input, providing a basis for prioritizing factors influencing molding quality. The outcomes establish a framework for optimizing process parameters and improving the overall performance of the cladding process.

## 4. Optimization of Algorithms and Process Parameters

### 4.1. Catch Optimization Fishing Algorithm (CFOA)

The Catch Fish Optimization Algorithm (CFOA) is a novel metaheuristic optimization technique inspired by farmers’ fishing practices in ponds. Known for its simplicity and efficiency, the algorithm was first proposed by Jia Heming [59]. The primary concept of CFOA focuses on maximizing yield through collective effort within a group. Individuals in the group utilize tools and exchange knowledge, allowing adaptation to complex aquatic environments. Shared experiences guide the group in identifying optimal fishing directions and locating areas with higher concentrations of fish.

**Figure 13:**
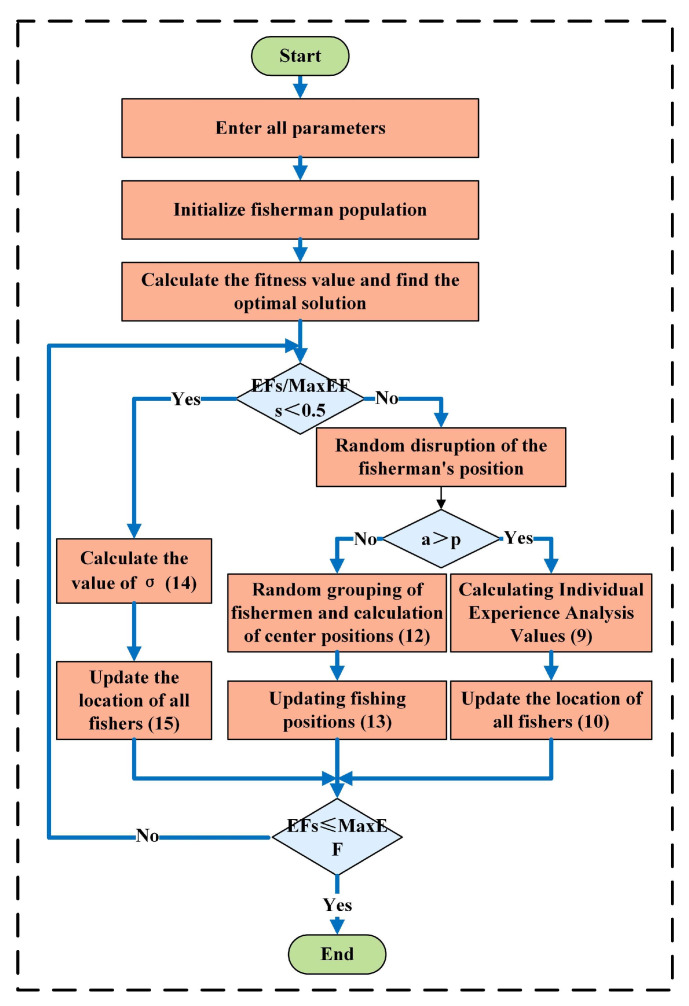
Algorithm logic diagram.

The CFOA algorithm is organized into several key steps, outlined in Figure 13. The process establishes a systematic framework for optimization, simulating iterative experience sharing and decision-making to achieve results in complex environments, as shown in the following:

(1) Initialization of the Population
  Solutions (“fishers”) are initialized within bounds (Equation (7)):
  (7) $$Fisher_{i,j}=\left(\begin{array}{c} ub_{j}-lb_{j} \end{array}\right)\times r+lb_{j}$$
(2) Exploration Phase
  Capture Rate α: (Equation (8)) balances exploration and exploitation:
  (8) $$\alpha ={\left(1-\frac{3\times EFs}{2\times EFs}\right)}^{\frac{3\times EFs}{2\times EFs}}$$

(i) Independent Search (*p* < *a*)
  Experiential analysis (Equation (9)):
  (9) $$Exp=\frac{fit_{i}-fit_{p}}{fit_{max}-fit_{min}}$$
  Search range adjustment (Equation (10)):
  (10) $$\;R=Dis\times \sqrt{\left|Exp\right|}\times \left(1-\frac{EFs}{MaxEFs}\right)$$
  Position update (Equation (11)):
  (11) $$Fisher_{i,j}^{T+1}=Fisher_{i,j}^{T}+\left(Fisher_{pos,j}^{T}-Fisher_{i,j}^{T}\right)\times Exp+r_{s}\times s\times R$$
(ii) Group Fishing (*p* > *a*)
  Group collaboration targets regions around centroids (Equation (12)):
  (12) $$Centre_{c}=mean(Fisher_{c}^{T})$$
  Positions updated via (Equation (13)):
  (13) $$Fisher_{c,i,j}^{T+1}=Fisher_{c,i,j}^{T}+r_{2}\times (Centre_{c}-Fisher_{c}^{T})+{\left(1-\frac{EFs}{MaxEFs}\right)}^{2}\times r_{3}$$

(3) Development Phase
  Gaussian refinement (Equations (14) and (15)) focuses on the global optimum:
  (14) $$\sigma =\sqrt{\left(2\left(1-\frac{\mathrm{EFs}}{\mathrm{MaxEFs}}\right)÷\left({\left(1-\frac{EFs}{MaxEFs}\right)}^{2}\right)+1\right)}$$
  (15) $$Fisher_{i}^{T+1}=Gbest+GD\left(0,\frac{r_{4}\times \sigma \times |mean(Fisher)-Gbest|}{3}\right)$$

### 4.2. Multi-Objective Analysis and Validation of Optimisation

The mathematical prediction model developed earlier is employed as the objective function for the CFOA optimization algorithm. To guarantee the feasibility of the optimization results and avoid generating unreasonable process parameters, practical engineering experience is incorporated by imposing specific constraints on the values of the three process parameters. The constraints are expressed in the following equation:

(16) $$\left\{\begin{array}{l} 900W\leq P\leq 2100W\\ 9mm\cdot s^{-1}\leq V_{s}\leq 14mm\cdot s^{-1}\\ 10g\cdot min^{-1}\leq V_{t}\leq 18g\cdot min^{-1} \end{array}\right.$$

The final form of the objective function is expressed as:

(17) $$\left\{\begin{array}{c} R=W\left(\begin{array}{c} f_{1}\\ f_{2}\\ f_{3} \end{array}\right)\\ 0\leq A\leq 5\\ 0\leq B\leq 6\\ 0\leq C\leq 5 \end{array}\right.$$

The CFOA optimization algorithm was applied to optimize the objective function *R.* Key parameters for the optimization process included a population size of 30, a maximum of 1000 iterations, and a random population group size between 3 and 4. Parameter values were chosen based on prior analysis and practical considerations. The optimization results identified an optimal solution of *A* = 3.4273, *B* = 1.8764, and *C* = 3.3667. In practical engineering terms, the corresponding process parameters were determined as *P* = 1628.19 W, *V_s_* = 9.9 mm/s, and *V_t_* = 14.73 g/min. The parameter combination was the optimal solution, balancing theoretical optimization with experimental feasibility. Experimental validation confirmed that the optimized fused cladding achieved a dilution rate of 0.353, microhardness of 744.64 HV, and an aspect ratio of 5.086. The measured response values for the optimized, control, and predicted groups are presented in Table 9. The results satisfied the experimental requirements and demonstrated the reliability of the optimization process.

**Figure 14:**
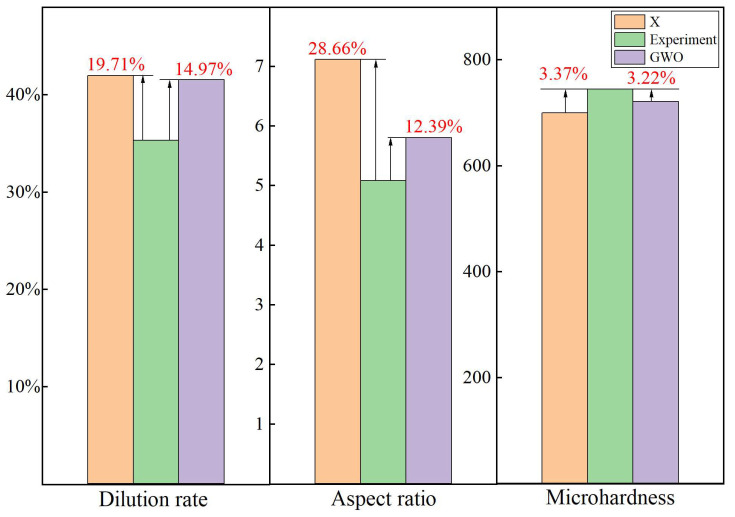
Target response value comparison graph.

The predicted values for the dilution rate, microhardness, and aspect ratio of the cladding layer closely matched the experimental results, with errors of 5.59%, 1.49%, and 0.17%, respectively, as depicted in Figure 14. Comparative experimental analysis indicated that utilizing the CFOA algorithm significantly improved key performance metrics: the cladding dilution rate was enhanced by 19.71%, microhardness increased by 3.37%, and the aspect ratio was enhanced by 28.66%. Comparative analysis with the GWO optimization algorithm demonstrated the superior performance of the CFOA-optimized cladding layer, achieving a 14.97% increase in cladding dilution rate, a 12.39% increase in aspect ratio, and a 3.22% increase in microhardness.

**Figure 15:**
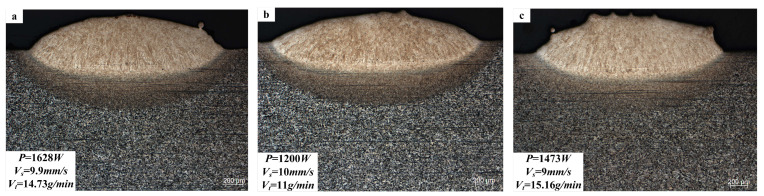
Cross-section of the fusion cladding layer: (**a**) optimal CFOA parameters; (**b**) engineering sample; (**c**) optimal GWO parameters.

Figure 15 indicates the cross-section of the fusion cladding layer under optimal CFOA parameters (a), engineering experimental sample (b), and the optimal GWO parameters (c). The distinct transition zones between the CFOA fusion cladding layer and the substrate reveal uniform microstructures and fine grains, indicating the overall performance. In contrast, the GWO-optimized group (Figure 15c) shows significant powder adhesion. Furthermore, the optimized process parameters ensured the absence of visible cracks and pores, demonstrating the fusion cladding layer’s high quality and structural integrity.

**Figure 16:**
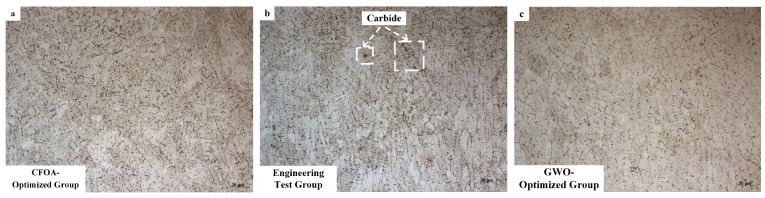
Microstructure of cross-section of cladding layers: (**a**) CFOA-optimized group; (**b**) GWO-optimized group; (**c**) engineering test group.

Figure 16 illustrates the cross-sectional microstructures of three distinct cladding layers: (a) the CFOA-optimized group, (b) the engineering test group, and (c) the GWO-optimized group. All three groups exhibited similar grain sizes and microstructural characteristics within their substrate regions.

The engineering test group (b), which was processed at 1200 W with insufficient heat input and low cooling rates, showed limited diffusion of alloying elements and carbon within the melt pool. This limitation resulted in localized carbon enrichment and the formation of granular carbides. These coarse carbides were concentrated at the grain boundaries, significantly compromising the material’s toughness and fatigue resistance.

In contrast, the CFOA-optimized group (a) employed medium-high power parameters in conjunction with a high-energy transient thermal cycle. This approach facilitated rapid heating and cooling transitions within the melt pool, effectively suppressing carbon diffusion and allowing a uniform dispersion of ultrafine carbide particles. The increased cooling rate also encouraged non-equilibrium solidification, leading to grain refinement.

The CFOA-optimized cladding layer exhibited an average hardness of 744.6 HV, representing a remarkable 3.2-fold increase compared to the substrate’s hardness of 232.2 HV. This improvement can be attributed to convection driven by the Marangoni effect, which helped homogenize thermal and chemical distributions while minimizing temperature gradients. Coupled with rapid surface cooling, the dynamic process accelerated solidification rates and facilitated the formation of fine equiaxed grains. The refinement of grains also increased the density of grain boundaries, enhancing resistance to dislocation motion through the Hall–Petch strengthening mechanism.

Overall, the optimization process significantly elevated the cladding layer’s quality. The CFOA algorithm exhibited exceptional precision in adjusting process parameters, offering a robust and innovative approach to advancing laser cladding technology. The findings provide critical insights for improving the efficiency and reliability of industrial laser cladding applications, paving the way for future advancements in material engineering.

## 5. Conclusions

This study explores the optimization of laser cladding technology for H13 die steel surfaces, introducing an innovative approach that combines a high-precision regression model with the Catch Fish Optimization Algorithm (CFOA) to achieve cladding performance. By systematically linking process parameters (laser power, scanning speed, and powder-feeding speed) with key performance metrics (dilution rate, microhardness, and aspect ratio), this research establishes a framework for advancing laser cladding optimization. The study identifies laser power as the most influential parameter and demonstrates significant improvements in cladding quality through multi-objective optimization, providing a robust foundation for practical applications.

(1) Development of a High-Precision Model: This study developed a reliable and adaptable regression model, with exceptional fitting accuracy based on experimental data. This model quantitatively reveals the relationships between process parameters and coating performance indicators, providing a theoretical foundation for optimizing laser cladding. Unlike conventional models, the approach uses systematic analysis with predictive capabilities, offering new insights into parameter interactions and their effects on cladding quality.
(2) Innovative Parameter Interaction Analysis: The analysis identified laser power as the most influential factor affecting the dilution rate while significantly influencing microhardness and aspect ratio. In addition, powder-feeding speed emerged as a critical determinant of aspect ratio, while scanning speed showed moderate effects but contributed notably through interactions with other parameters, particularly laser power. Identifying these intricate interactions, especially between laser power and scanning speed, highlight the importance of multi-parameter optimization in achieving consistent and high-quality cladding layers.
(3) Integration of CFOA for Multi-Objective Optimization: The CFOA algorithm was applied within a multi-objective optimization framework, where the analytic hierarchy process (AHP) transformed a complex multi-objective problem into a single-objective optimization. This innovative integration enabled the precise determination of optimal process parameters: laser power of 1628 W, scanning speed of 9.9 mm/s, and powder-feeding speed of 14.7 g/min. The optimized parameters achieved a dilution rate of 0.353, Vickers hardness of 744.64 HV, and an aspect ratio of 5.086, with prediction errors remaining below 6%. The level of precision marks a significant advancement over conventional optimization techniques.
(4) Performance Improvements Achieved Through Optimization: The optimized cladding layer achieved substantial improvements, including a 19.71% increase in dilution rate, a 3.37% rise in microhardness, and a 28.66% enhancement in aspect ratio. Compared to conventional optimization methods, and outperforming the GWO algorithm with a 14.97% higher dilution rate, a 12.39% higher aspect ratio, and a 3.22% increase in microhardness, the CFOA algorithm demonstrated global search capabilities, enabling efficient exploration of the parameter space and delivering a more accurate and practical solution. The proposed methodology offers an innovative solution to tackle the complexities of multi-objective optimization in laser cladding.

This study establishes a novel method for optimizing laser cladding, integrating high-precision regression models with advanced algorithms like CFOA. The findings improve understanding of parameter interactions and their impact on cladding performance. Future work could explore its application to other materials and processes and hybrid optimization techniques for enhanced adaptability.

## Figures and Tables

**Table 1:** Chemical composition of H13 die steel powder.

Composition	C	Si	Mn	Cr	Mo	V	P	S
Wt/%	0.35~0.45	0.80~1.20	0.20~0.50	4.75~5.50	1.10~1.75	0.80~1.20	≤0.03	≤0.03

**Table 2:** Orthogonal experiment factors and levels.

Level	P/W	$V_{s}/\left(mm\cdot s^{-1}\right)$	$V_{t}/\left(g\cdot {min}^{-1}\right)$
1	900	9	10
2	1200	10	12
3	1500	11	14
4	1800	12	16

**Table 3:** Experimental design and measured target response values.

No.	P/W	$V_{s}/\left(mm\cdot s^{-1}\right)$	$V_{t}/\left(g\cdot {min}^{-1}\right)$	η/%	HV	W/H
1	900	9	10	0.70	720.00	15.79
2	900	10	12	0.53	637.93	8.75
3	900	11	14	0.54	703.63	9.54
4	900	12	16	0.49	714.67	10.00
5	1200	9	12	0.42	686.30	6.31
6	1200	10	10	0.48	719.90	8.13
7	1200	11	16	0.38	731.07	6.71
8	1200	12	14	0.43	753.03	7.89
9	1500	9	14	0.30	748.63	6.32
10	1500	10	16	0.25	725.67	4.68
11	1500	11	10	0.42	746.73	7.29
12	1500	12	12	0.43	731.27	7.97
13	1800	9	16	0.45	712.43	7.25
14	1800	10	14	0.36	719.00	5.63
15	1800	11	12	0.41	732.27	7.89
16	1800	12	10	0.46	740.10	8.84

**Table 4:** Standardized data for response values.

No.	η	HV	W/H
1	1	0.2870	1.000
2	0.575	1.0000	0.366
3	0.6	0.4292	0.437
4	0.475	0.3333	0.479
5	0.3	0.5798	0.147
6	0.45	0.2879	0.310
7	0.2	0.1908	0.182
8	0.325	0.0000	0.289
9	0	0.0382	0.147
10	0.125	0.2378	0.000
11	0.3	0.0547	0.234
12	0.325	0.1891	0.296
13	0.375	0.3527	0.231
14	0.15	0.2957	0.086
15	0.275	0.1804	0.289
16	0.4	0.1124	0.374

**Table 5:** ANOVA of the prediction model for dilution rate.

Source	DOF	Seq SS	Adj SS	Adj MS	F	*p*
Model	11	0.804375	0.804375	0.073125	105.46	<0.0001
A	1	0.297070	0.000779	0.000779	1.12	0.349
B	1	0.001758	0.028970	0.028970	41.78	0.003
C	1	0.138195	0.017377	0.017377	25.06	0.007
$A^{2}$	1	0.208164	0.002234	0.002234	3.22	0.147
$B^{2}$	1	0.017227	0.025025	0.025025	36.09	0.004
$C^{2}$	1	0.037539	0.037539	0.037539	54.14	0.002
A×B	1	0.002472	0.003751	0.003751	5.41	0.081
A×C	1	0.075018	0.000145	0.000145	0.21	0.671
$B^{3}$	1	0.017053	0.022563	0.022563	32.54	0.005
$A^{2}\times B$	1	0.006328	0.006328	0.006328	9.13	0.039
$A^{2}\times C$	1	0.003551	0.003551	0.003551	5.12	0.086
Inaccuracies	4	0.002773	0.002773	0.000693		
Total	15	0.807148				

**Table 6:** ANOVA of the prediction model for microhardness.

Source	DOF	Seq SS	Adj SS	Adj MS	F	*p*
Model	13	0.869218	0.869218	0.066863	10.35	0.091
A	1	0.186592	0.002680	0.002680	0.41	0.585
B	1	0.100460	0.124499	0.124499	19.28	0.048
C	1	0.000058	0.282711	0.282711	43.78	0.022
$A^{2}$	1	0.124679	0.000369	0.000369	0.06	0.833
$B^{2}$	1	0.038412	0.093964	0.093964	14.55	0.062
$C^{2}$	1	0.045772	0.208909	0.208909	32.35	0.030
A×B	1	0.004208	0.018436	0.018436	2.85	0.233
A×C	1	0.000006	0.026388	0.026388	4.09	0.181
B×C	1	0.063425	0.079572	0.079572	12.32	0.072
$A^{3}$	1	0.019368	0.019368	0.019368	3.00	0.225
$B^{3}$	1	0.071608	0.086879	0.086879	13.45	0.067
$C^{3}$	1	0.194022	0.194022	0.194022	30.04	0.032
$A^{2}\times B$	1	0.020607	0.020607	0.020607	3.19	0.216
Inaccuracies	2	0.012916	0.012916	0.006458		
Total	15	0.882133				

**Table 7:** ANOVA of the prediction model for aspect ratio.

Source	DOF	Seq SS	Adj SS	Adj MS	F	*p*
Model	13	0.750623	0.750623	0.057740	21.07	0.046
A	1	0.215749	0.062103	0.062103	22.66	0.041
B	1	0.000170	0.018067	0.018067	6.59	0.124
C	1	0.129407	0.044355	0.044355	16.18	0.057
$A^{2}$	1	0.171351	0.028187	0.028187	10.28	0.085
$B^{2}$	1	0.069978	0.020626	0.020626	7.53	0.111
$C^{2}$	1	0.035597	0.012699	0.012699	4.63	0.164
A×B	1	0.006587	0.006918	0.006918	2.52	0.253
A×C	1	0.033954	0.056039	0.056039	20.45	0.046
B×C	1	0.017262	0.019367	0.019367	7.07	0.117
$A^{3}$	1	0.011295	0.020177	0.020177	7.36	0.113
$B^{3}$	1	0.040979	0.040979	0.040979	14.95	0.061
$C^{3}$	1	0.009411	0.009411	0.009411	3.43	0.205
$A\times B^{2}$	1	0.008884	0.008884	0.008884	3.24	0.214
Inaccuracies	2	0.005481	0.005481	0.002741		
Total	15	0.756104				

**Table 8:** AHP judgment matrix.

Formation Quality	Dilution Rate	Microhardness	Aspect Ratio
Dilution Rate	1	3/4	6/5
Microhardness	4/3	1	4/3
Aspect Ratio	5/6	3/4	1

**Table 9:** Response measurements of the optimized, control, and predicted groups.

Parameter	Predicted Value	CFOA-Optimized Group	GWO-Optimized Group	Engineering Test Group
Dilution Rate (η)	0.335	0.353	0.416	0.440
Microhardness (HV)	743.360	744.640	721.435	720.350
Aspect Ratio (W/H)	5.011	5.086	5.805	7.128

## Data Availability

The original contributions presented in the study are included in the article, further inquiries can be directed to the corresponding author.
